# Glucomannan Accumulation Induced by Exogenous Lanthanum in *Amorphophallus konjac*: Insights from a Comparative Transcriptome Analysis

**DOI:** 10.3390/biology14070849

**Published:** 2025-07-11

**Authors:** Xiaoxian Li, Zhouting Zeng, Siyi Zhu, Xirui Yang, Xiaobo Xuan, Zhenming Yu

**Affiliations:** 1 School of Pharmaceutical Sciences, Zhejiang Chinese Medical University, Hangzhou 310053, China; lixiaoxian@zcmu.edu.cn (X.L.);; 2 The First Affiliated Hospital of Zhejiang Chinese Medical University, Hangzhou 310006, China

**Keywords:** *Amorphophallus konjac*, konjac glucomannan, lanthanum, transcriptome

## Abstract

Konjac glucomannan (KGM), derived from *Amorphophallus konjac*, is increasingly utilized in food and pharmaceutical applications. However, inconsistent KGM production across cultivars jeopardizes its quality and market viability. Lanthanum (La) has been shown to promote KGM levels, but the underlying mechanism remains unclear. In this study, 20~80 mg L^−1^ La significantly stimulated KGM accumulation compared with the control group. We performed a transcriptome analysis and found 21,047 differentially expressed genes (DEGs), predominantly enriched in carbohydrate and glycan metabolism pathways. A total of 48 DEGs were linked to KGM biosynthesis, with 20 genes (*SuSy*, *INV1/3/5/6*, *HK1/2*, *FPK2*, *GPI3*, *PGM3*, *UGP2*, *GMPP1/4*, *CslA3~7*, *CslH2*, and *MSR1.2*) showing significant positive correlations with KGM content. Interestingly, three key terminal pathway genes (*UGP1*, *UGP3*, and *CslD3*) exhibited strong upregulation (log2 fold change > 3). Seven DEGs were validated with qRT-PCR, aligning with the transcriptomic results. Furthermore, 12 hormone-responsive DEGs, including 4 ethylene-related genes (*CTR1*, *EBF1/2*, *EIN3*, and *MPK6*), 6 auxin-related genes (*AUX/IAA1-3*, *SAUR1-2*, and *TIR1*), and 2 gibberellin-related genes (*DELLA1-2*), were closely linked to KGM levels. Additionally, the transcription factors bHLH and AP2/ERF showed to be closely related to the biosynthesis of KGM. These results lay the foundation for a model wherein La (Ш) modulates KGM accumulation by coordinately regulating biosynthetic and hormonal pathways via specific transcription factors.

## Simple Summary

Konjac glucomannan (KGM), a valuable polysaccharide from *Amorphophallus konjac*, is important in the food and medical fields, but its production varies across cultivars, which affects its quality and market viability. Lanthanum (La) can cause an increase in KGM levels, but the mechanisms underlying this effect remain unclear. Here, we found that La (20~80 mg L^−1^) increased KGM content and altered gene activity, especially in pathways related to carbohydrate metabolism and hormone signaling. Key genes for KGM synthesis (*Csls*, *UGP*, etc.) and hormone responses were linked to KGM levels. Our results suggest that La improves KGM production by regulating both biosynthesis genes and hormone signals, laying the foundation for new methods to enhance *A. konjac* quality.

## 1. Introduction

*Amorphophallus konjac* is a perennial, herbaceous monocot belonging to the genus *Amor-phophallus* in the Araceae family [1,2] which grows in tropical and subtropical regions particularly in Asia, such as China, Myanmar, Vietnam, and Indonesia. About 170 species of Konjac have been identified in the world, and of the 21 species that have been discovered and named in China, 9 are endemic to the country [3]. Konjac has been used and cultivated as a traditional medicine and a food source for more than 2000 years in China [4,5]. Konjac glucomannan (KGM), a natural polysaccharide derived from the konjac corm, has been widely used in agriculture, chemistry, medicine, and other industries because of its strong water absorption, high swelling rate, and good gelling and film-forming properties [6,7,8]. Moreover, in 2021, KGM was approved by the FDA as a food additive in the treatment of obesity-related dyslipidemia and diabetes due to its effectiveness in lowering blood cholesterol and sugar levels and promoting intestinal activity and immune function [9].

With the increasingly greater demand and economic value of KGM, konjac cultivation is important to agricultural industries and farmers’ prosperity in China. The expansion of cultivation areas, continuous cropping, and inadequate agronomic measures have led to low propagation coefficients and yield, high sensitivity to diseases, and declines in KGM contents, resulting in a deterioration in quality [10]. Hence, the investigation of the regulatory network related to KGM biosynthesis for the improvement in the quality of cultivated konjac is urgently needed. 

Lanthanum, La (Ш), one of the primary components of rare-earth micro-fertilizers, has been defined as a beneficial element on account of its effectiveness in promoting crop yield and improving crop quality [11,12]. It is reported that La (Ш) is conducive to the uptake of K, Ca, and Mg; promotes root and whole-plant growth; improves germination, especially via the signaling pathway mediated by calmodulin; and activates antioxidants [11,13]. Additionally, La (Ш) alleviates various types of abiotic stress, for instance, those due to heavy metals [14], high salt concentrations [15], and ultraviolet radiation [16]. Similar to other rare-earth elements [17,18], La (Ш) exhibits a hormone-like, biphasic dose–response effect on plant growth and development, characterized by low-dose stimulation and high-dose inhibition [12]. The maximum concentrations for plant growth vary significantly among different species; for instance, 81.6 μM is optimum for stomatal conductance and growth in *Oryza sativa* [19], while 35 μM is optimum for chlorophyll and growth in *Armoracia rusticana* [20], and 100 mg/L La was shown to significantly promote growth in Chinese cabbage [21]. Our preliminary research study found that 20~160 mg L^−1^ La (Ш) increased KGM content in *A. sinensis* [12]. However, the genes involved in KGM biosynthesis under exposure to La (Ш) have not yet been determined.

Building upon our previous research work, the effects of different La (Ш) concentrations on the expression levels of KGM biosynthetic genes in *A. konjac* were investigated with transcriptome sequencing. The differential accumulation of KGM and its metabolic pathways under treatment with La (Ш) in *A. konjac* were also determined. Additionally, the regulatory network related to KGM biosynthesis with or without La (Ш) was investigated. The findings provide a genetic foundation for breeding high-KGM konjac and offer valuable insights for La (Ш) application in the cultivation of commercial plants.

## 2. Materials and Methods

### 2.1. Plant Materials

The one-year-old *A. konjac* corms used in the experiment were purchased in Qujing City, Yunnan Province, and authenticated by Professor Shuili Zhang from Zhejiang Traditional Chinese Medicine University as *A. konjac*. Similar-weight corms (approximately 100 g) were selected and planted at a planting density of 100 cm × 100 cm in the plantation of Zhejiang Traditional Chinese Medicine University (30°5′26″ N, 119°53′40″ E) in April 2022. The experimental site is characterized by a subtropical monsoon climate with loam soil (pH 6.5~7.5), and field management (irrigation, insect, and weed control) was implemented according to weather and local agronomic practices.

### 2.2. La (Ш) Treatment

In the early stages of leaf expansion (namely, the early stage of corm expansion), solutions with three concentrations of La (Ш) (20, 80, and 160 mg L^−1^) were evenly sprayed on the leaves of *A. konjac*. The La (Ш) solutions were prepared with appropriate quantities of LaCl_3_·7H_2_O (Sigma-Aldrich, Corporation, St. Louis, MO, USA) and distilled water. Additionally, the same amount of distilled water, where the concentration of La (Ш) was 0, was used for the control (CK) group. Following a single application of La (Ш), *A. konjac* corms were collected after 7 days (with three biological replicates) and immediately flash-frozen in liquid nitrogen for stabilization, followed by storage at −80 °C prior to RNA extraction.

### 2.3. Determination of Konjac Glucomannan (KGM) Content

The *A. konjac* corms were harvested 14 and 60 days post-treatment following a single application of La (Ш) and oven-dried at 80 °C to constant weight. The content of KGM was calculated based on the 3,5-dinitrosalicylic acid assay [22].

### 2.4. RNA Extraction, cDNA Library Construction, and Transcriptome Sequencing

Total RNA from each sample with or without La (Ш) treatment was extracted using a Quick RNA isolation Kit (Huayueyang Biotechnology Co., Beijing, China) based on the manufacturer’s protocol. Prior to library construction, RNA quality, quantity, and integrity were assessed using Nanodrop 2000 spectrophotometry (Thermo Fisher Scientific Inc., Waltham, MA, USA), Qubit 2.0 fluorometry (Thermo Fisher Scientific Inc., Waltham, MA, USA), and the Agilent 2100 Bioanalyzer (Agilent Technologies Inc., Santa Clara, CA, USA). Triplicates of each treatment were used for RNA sequencing; then, the samples were concentrated using oligo (dT) magnetic adsorption. The concentrated samples served as templates for the synthesis of first-strand cDNA by using random hexamers and reverse transcriptase. Second-strand cDNA was synthesized and purified using AMPure XP (A63881, Beckman Coulter, Inc., Brea, CA, USA) beads and resolved in EB buffer for poly(A) and adapter addition. cDNA fragments of suitable lengths and insert sizes were selected with the AMPure XP beads to construct the final cDNA libraries. The cDNA libraries were checked using Qubit 2.0 and Agilent 2100 before they were sequenced using the Illumina sequencing platform. Then, 12 libraries were sequenced in an Illumina Hiseq 2500 system at Genepioneer Biotechnologies Co, Ltd., Nanjing, China (http://www.genepioneer.com/, accessed on 1 December 2022). For each end of the produced paired-end sequences, 150 bases were sequenced. The transcript abundance values were quantified and normalized using the FPKM (fragments per kilobase of exon per million mapped fragments) method. Pearson correlation analyses were carried out on variance-stabilized transformed values.

### 2.5. Assembly, Data Analysis, and Functional Annotation

The raw reads were first subjected to quality control by using FastQC (v0.11.9). Adapter sequences and low-quality reads (>5% ambiguous bases) were trimmed and filtered using Trimmomatic (v0.39). The clean reads from all samples were pooled and assembled de novo by using Trinity (v2.11.0) for the construction of a candidate unigene library for the target species. The assembly generated 688,528 transcripts and 374,834 unigenes, with N50 values of 1707 bp and 1200 bp, respectively. Clean reads from each biological replicate were mapped to the reference transcriptome by using TopHat 2.0.11 (with default parameters). The transcriptomes of the *A. konjac* samples treated with 0, 20, 80, and 160 mg L^−1^ La (Ш) were reconstructed using Cufflinks 2.2.1 (with default parameters). The assemblies were merged using the Cuffmerge module to generate a unified transcriptome for downstream expression analysis. For functional annotation, all unigenes were searched against 6 functional protein databases, i.e., the NCBI non-redundant protein database (Nr; ftp://ftp.ncbi.nlm.nih.gov/blast/db/, accessed on 1 December 2022), Clusters of Orthologous Groups (COG; http://www.ncbi.nlm.nih.gov/COG/, accessed on 1 December 2022), the Gene Ontology database (GO; http://www.geneontology.org, accessed on 1 December 2022), the Kyoto Encyclopedia of Genes and Genomes (KEGG; http://www.genome.jp/kegg, accessed on 1 December 2022), Pfam (http://pfam.xfam.org/, accessed on 1 December 2022), and Swiss-Prot (https://www.expasy.org/resources/uniprotkb-swiss-prot, accessed on 1 December 2022), using BLASTX alignment with an E-value cut-off of 10^−5^. Additionally, transcription factors (TFs) were identified using the Plant Transcription Factor Database (PlantTFDB; http://planttfdb.cbi.pku.edu.cn/, accessed on 1 December 2022).

### 2.6. Analysis of Differentially Expressed Genes

The FPKM values of the differentially expressed genes (DEGs) were calculated based on the normalized reads by using the DESeq R Package (v1.26.0) in Bioconductor (http://www.bioconductor.org/, accessed on 2 December 2022), and significantly DEGs among different samples were identified according to the following criteria: *p* < 0.05 and |log2 fold change (log2 FC)| > 1 [23].

### 2.7. Protein–Protein Interaction (PPI) Network Analysis of KGM Biosynthetic Enzymes and TFs

La (Ш)-responsive TFs potentially involved in KGM biosynthesis regulation were identified with BLASTP analysis against *Arabidopsis thaliana* (E-value < 1 × 10^−5^). PPI networks were constructed with known KGM biosynthetic enzymes and TF proteins by using STRING (confidence score > 0.7), followed by visualization and analysis using Cytoscape (v3.9.1).

### 2.8. Quantitative Real-Time PCR (qRT-PCR) Verification

We verified the quality of the RNA-Seq data by randomly selecting 7 DEGs with *Elongation factor-1 alpha* (*EF-1α*) as the internal reference gene [24] and designing specific primers with the PrimerQuest Tool (https://sg.idtdna.com/PrimerQuest/Home/Index, accessed on 5 March 2023); these primers were then synthesized at Biotechnology Co., Shanghai, China, as shown in Table S1. Full-length cDNA was synthesized using the PrimeScript™ RT reagent Kit with a gDNA Eraser (Perfect Real Time) kit (RR047A, Takara, Dalian, China). According to the procedure outlined, 0.5 μL of cDNA (2.5 ng μL^−1^), 5 μL of 2 × iTaq™ SYBR^®^ Green Supermix (Bio-Rad Laboratories, Hercules, CA, USA), 0.75 μL of upstream and downstream primers, and 3 μL of ddH_2_O were mixed for qRT-PCR detection with a CFX96 Real-Time PCR instrument (Bio-Rad). The qRT-PCR procedure was as follows: pre-denaturation at 95 °C for 3 min, 40 cycles of denaturation at 95 °C for 5 s, and annealing at 60 °C for 30 s. The relative expression of each gene was calculated using the 2^−ΔΔCT^ protocol. Three biological replicates were performed per sample.

### 2.9. Statistical Analysis

The data analyses were carried out with SPSS 25.0 (IBM Co., Armonk, NY, USA). One-way analysis of variance (ANOVA) and Duncan’s multiple range test (DMRT) were used for the significance analysis of different La (Ш) treatments. Data normality was verified using the Shapiro–Wilk test (*p >* 0.05 for all variables). Intergroup correlations were then analyzed using the Pearson correlation coefficient, with statistical significance set to *p <* 0.05.

## 3. Results

### 3.1. KGM Content in Plants Exposed to Different La (Ш) Concentrations

**Figure 1:**
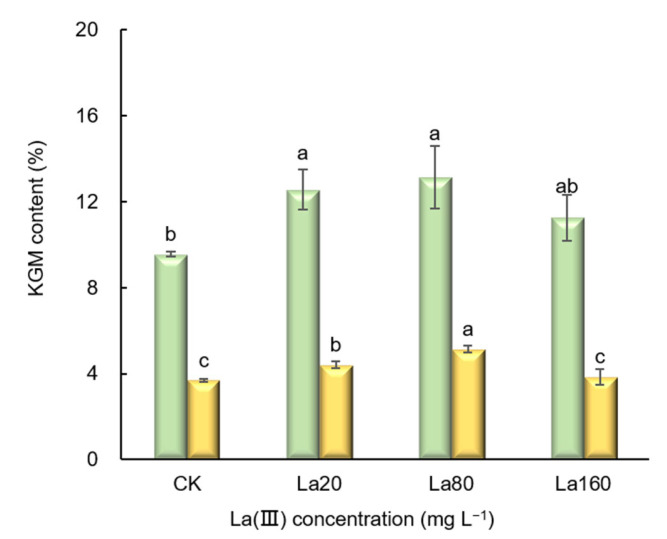
KGM content 14 days (yellow color) and 60 days (green color) after La (Ш) treatment (means ± SD, *n* = 3). CK: control group. La20: 20 mg·L^−1^ La (Ш) concentration. La80: 80 mg·L^−1^ La (Ш) concentration. La160: 160 mg·L^−1^ La (Ш) concentration. Different lowercase letters indicate significant differences between treatments (Duncan’s multiple range *p* < 0.05).

Building on previous findings showing that foliar La (Ш) application (20~160 mg L^−1^) enhances konjac glucomannan (KGM) accumulation in *A. sinensis* [12], in this study, we performed a single foliar application of La (Ш) at varying concentrations (20~160 mg L^−1^) during the leaf expansion stage of *A. konjac*. The KGM content was subsequently quantified 14 and 60 days post-treatment. We observed that 14 days post-treatment, compared with CK, the KGM content increased following different La (Ш) treatments; the highest values were observed under the 20 mg L^−1^ and 80 mg L^−1^ treatments, where the content increased by 19.48% and 39.13% (*p <* 0.05), respectively. Similarly, 60 days post-treatment, compared with CK, the KGM content increased following different La (Ш) treatments; the highest values were again observed under the 20 mg L^−1^ and 80 mg L^−1^ treatments, where the content increased by 28.03% and 37.47% (*p <* 0.05), respectively (Figure 1 and Table S2).

### 3.2. Assembly and Quality Assessment of Transcriptome Sequencing

Fresh *A. konjac* corms were collected 7 days after being treated with La (Ш) in three replicates and were used to extract the total RNA and construct 12 libraries for RNA-Seq. As shown in Table 1, 5.93–9.51 Gb of raw sequencing reads was generated, with the average raw reads being 7.24 Gb. After quality filtering, a range of 15.83 to 25.11 Mb of clean reads was obtained, with an average of 19.68 Mb of clean reads. The average GC content was 51.55%, and the average Q30 value was 92.23%. These results indicate that the transcriptome data of *A. konjac* corms under different La (Ш) treatments were of high quality.

### 3.3. Functional Annotation and Classification of Unigenes

**Figure 2:**
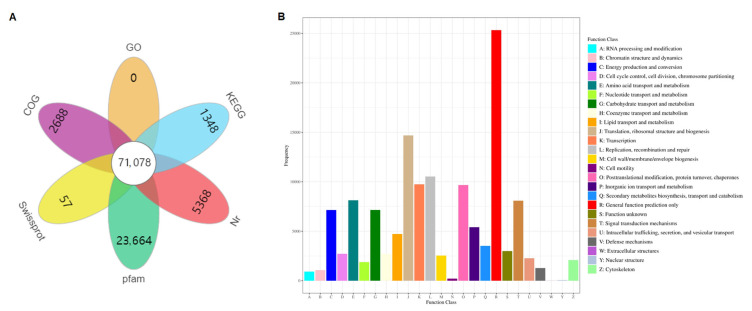
De novo transcriptome assembly and annotation. (**A**) Venn diagram of functional annotation of 6 public protein databases (COG, GO, KEGG, Pfam, Swiss-Prot, and Nr). (**B**) COG enrichment analysis of all unigenes.

A total of 231,443 unigenes were annotated based on the six public protein databases considered, with 100,765 unigenes for COG, 142,047 for GO, 181,135 for KEGG, 163,578 for Pfam, 149,376 for Swiss-Prot, and 189,287 for the Nr database (Table S3). Out of the total, 71,078 unigenes in *A. konjac* significantly matched those from the above six public protein databases. In addition, the assembled unigenes in the Nr, COG, Pfam, Swiss-Prot, and KEGG databases contained 5368, 2688, 23,664, 57, and 1348 unigenes, respectively, and no unigenes were annotated in the GO database (Figure 2A). Moreover, the 100,765 unigenes form the COG database were divided into 25 COG categories, where the 5 most annotated groups were “general function prediction only” (25,329), “translation, ribosomal structure and biogenesis” (14,687), “replication, recombination and repair” (10,528), “transcription” (9737), and “posttranslational modification, protein turnover, chaperones” (9667) (Figure 2B).

### 3.4. Analysis of Differentially Expressed Genes (DEGs)

**Figure 3:**
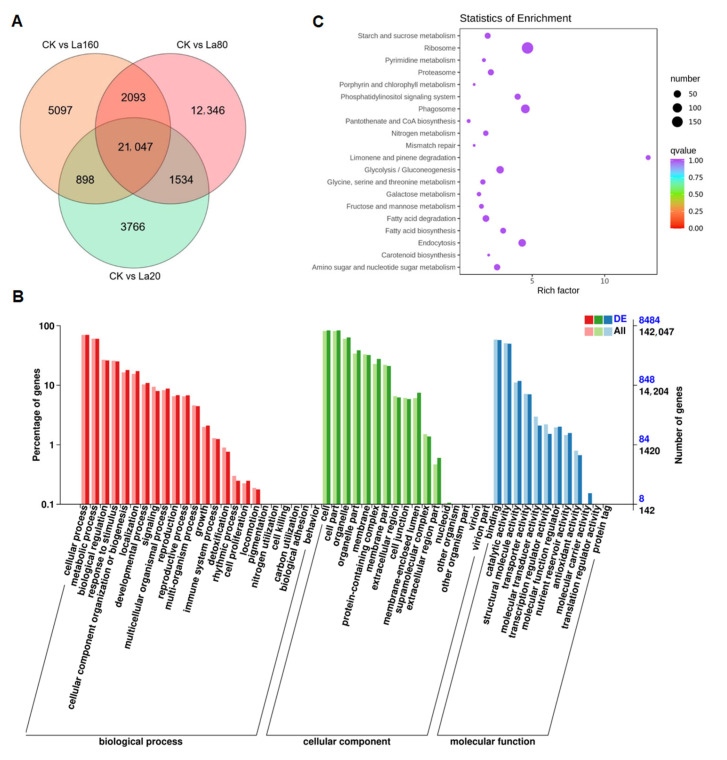
Differentially expressed genes (DEGs). (**A**) Venn diagram depicting number and overlap among DEGs for each combination (CK vs. La20, CK vs. La80, and CK vs. La160). CK: control group. La20: 20 mg·L^−1^ La (Ш) concentration. La80: 80 mg·L^−1^ La (Ш) concentration. La160: 160 mg·L^−1^ La (Ш) concentration. (**B**) GO enrichment of DEGs. (**C**) Top 20 KEGG enriched DEGs for the three combinations.

Three treatment combinations (CK vs. La20, CK vs. La80, and CK vs. La160) were considered for the investigation of the DEGs among treatments with different concentrations of La (Ш) (Figure 3A). Compared with CK, CK vs. La80 showed the highest number of DEGs (37,020) and CK vs. La20 the lowest (27,245). The three combinations shared 21,047 common DEGs. Moreover, CK vs. La20, CK vs. La80, and CK vs. La160 showed 3766, 12,346, and 5097 unique DEGs, respectively, indicating that some genes may be silenced under different La (Ш) treatments.

The 21,047 common DEGs of the three combinations were used for GO and KEGG enrichment analyses. In GO enrichment, the DEGs were mainly annotated to “cell” and “cell part” for “Cellular Component” (Figure 3B), “binding” and “catalytic activity” for “Molecular Function”, and “cellular process” and “metabolic process” for “Biological Process”. The KEGG enrichment analysis showed that the DEGs of the three combinations were enriched in 126 pathways (Figure 3C). Both “propanoate metabolism” (map00640) and “pentose and glucuronate interconversions” (map00040) were associated with carbohydrate metabolism, while “N-Glycan biosynthesis” (map00510) and “glycosyl-phosphatidylinositol (GPI)-anchor biosynthesis” (map00563) were related to glycan biosynthesis and metabolism.

### 3.5. Identification of KGM Biosynthetic Genes in A. konjac

**Figure 4:**
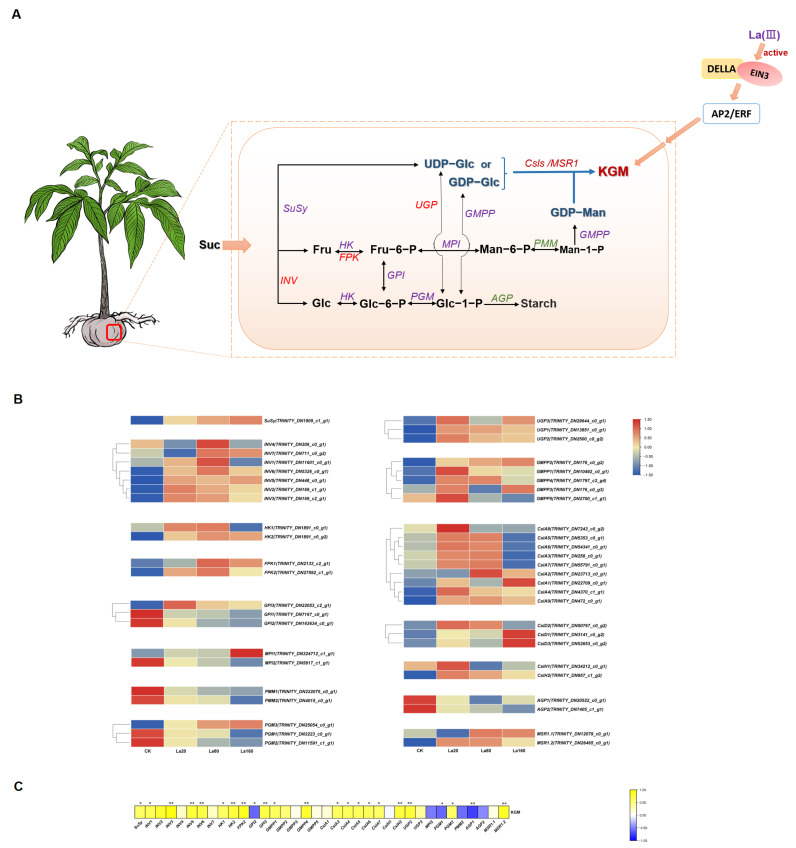
Biological pathway of KGM and its DEG heatmap. (**A**) Biological pathway of KGM biosynthesis. Sucrose (Suc), fructose (Fru), glucose (Glc), GDP-mannose (GDP-man), UDP-glucose (UDP-Glc), fructose-6-phosphate (Fru-6-P), fructose-1-phosphate (Fru-1-P), mannose-6-phosphate (Man-6-P), mannose-1-phosphate (Man-1-P), glucose-6-phosphate (Glc-6-P), and glucose-1-phosphate (Glc-1-P). Sucrose synthase (*SuSy*), invertase (*INV*), phosphoglucose isomerase (*PGI*), phosphoglucomutase (*PGM*), phosphomannose isomerase (*PMI*), phosphomannomutase (*PMM*), GDP-mannose pyrophosphorylase (*GMPP*), UDP-glucose pyrophosphorylase (*UGP*), ADP-glucose pyrophosphorylase (*AGP*), fructokinase (*FRK*), hexokinase (*HK*), cellulose synthase-like (*CSLs*), and mannan synthesis-related 1(*MSR1*). (**B**) Heatmap illustrating the expression profiles of the 48 DEGs in the KGM biological pathway. FPKM values visualized using logarithmic transformation and row-wise normalization. Color scale represents relative expression levels (blue: low; red: high). CK: control group. La20: 20 mg·L^−1^ La (Ш) concentration. La80: 80 mg·L^−1^ La (Ш) concentration. La160: 160 mg·L^−1^ La (Ш) concentration. (**C**) Heatmap of Pearson correlation between FPKM values of DEGs and konjac glucomannan (KGM) content. Among the 48 DEGs analyzed, 36 showed a normal distribution (Shapiro−Wilk test, *p* > 0.05) and were included in the Pearson correlation analysis. Color scale represents relative expression levels (blue: low; yellow: high). * *p* < 0.05 and ** p < 0.01 (Pearson correlation).

KGM biosynthesis was found to be closely related to the carbon metabolism pathway. Sucrose (Suc), the primary product of photosynthesis, is transported to the corm by the phloem and is catalyzed into GDP-mannose (GDP-man) and UDP-glucose (UDP-Glc) through a series of phosphorylation processes; finally, KGM is biosynthesized under the effect of cellulose-like synthases (Csls) [25], as shown in Figure 4 and Table S4. A total of 48 genes were annotated to 15 enzymes in the KGM biosynthetic pathway under La (Ш) treatment (Figure 4A). One gene (*SuSy*) was annotated to Suc synthase, which catalyzes the reversible cleavage of Suc into fructose (Fru) and UDP-Glc. Seven genes were annotated to invertase (*INV*), which catalyzes Suc’s conversion into glucose (Glc) and Fru. Two genes were annotated to fructokinase (*FPK*), which catalyzes the reversible conversion between Fru and fructose-6-phosphate (Fru-6-P). Two genes were found to encode hexokinase (*HK*), which exhibits the same catalytic activity as *FPK*. Three genes were found to encode UGP and five genes GDP-mannose pyrophosphorylase (*GMPP*), both of which are responsible for the KGM precursors UGP-Glc/GDP-Glc and GDP-Man, respectively. Two genes were annotated to mannan synthesis-related 1 (*MSR1*). Ten genes were annotated to four major classes of phosphorylated isomerases: three genes to glucose-6-phosphate (Glc-6-P) isomerase (*GPI*), three genes to phosphoglucomutase (*PGM*), two genes to mannose-6-phosphate (Man-6-P) isomerase (*MPI*), and two genes to phosphomannomutase (*PMM*). A total of 14 genes were annotated to cellulose-like synthases (*Csls*). Among them, nine genes were annotated to *CslA*, three genes to *CslD*, and two genes to *CslH*.

The FPKM values of the *SuSy* and *INV5* genes increased under different concentrations of La (Ш) (20 to 160 mg L^−1^), with both reaching higher levels under 160 mg L^−1^ La (Ш), while the FPKM values of other *INVs* initially increased and then decreased with the increase in the concentration of La (Ш). The expression levels of *INV1/4/6/7* were higher under 80 mg L^−1^ La (Ш) (*p* < 0.05), and those of *INV2/3* were higher under 20 mg L^−1^ La (Ш) (*p <* 0.05). Additionally, two genes were annotated to *FPKs*, which catalyze the reversible conversion between Fru and Fru-6-P. Two genes encoded *HKs*, which have the same catalytic activity as *FPKs* but also catalyze the reversible conversion between Glc and Glc-6-P. Similar to the trend in *INV7* expression*,* the expression levels of *HK1/2* and *FPK1/2* increased firstly and then decreased with the increase in the concentration of La (Ш), peaking under 80 mg L^−1^ La (Ш), and significant differences were observed in the expression of *FPK2*.

Genes annotated to *UGP* and *GMPP* displayed differential responses to the different concentrations of La (Ш). *UGP1/2/3* and *GMPP1/5* showed significantly higher FPKM values under 20 mg L^−1^ La (Ш) (*p <* 0.05), and *GMPP 4* showed significantly higher FPKM values under both 20 mg L^−1^ and 80 mg L^−1^ (*p <* 0.05), while *GMPP2* increased progressively under concentrations from 20 to 160 mg L^−1^ La (Ш), peaking at 160 mg L^−1^ (*p <* 0.05). Conversely, *GMPP3* showed lower expression under 80 mg L^−1^ La (Ш) but higher expression under 20 mg L^−1^ and 160 mg L^−1^ La (Ш) (*p <* 0.05). Meanwhile, the FPKM values of *GPI3*, *MPI1*, and *PGM3* were higher than the control (CK) under different concentrations of La (Ш). *GPI3* and *MPI1* exhibited higher expression under 20 mg L^−1^ and 160 mg L^−1^, respectively (*p* < 0.05). *PGM3* had higher expression under 80 mg L^−1^ and 160 mg L^−1^ La (Ш) (*p* < 0.05). Conversely, compared with CK, the FPKM values of *MPI2*, *PMM1/2*, *PGM1/2,* and *GPI1/2* showed a downward trend with the increase in the concentration of La (Ш).

Csls, belonging to the glycosyltransferase GT2 family, are key enzymes in glycan synthesis [26]. La (Ш) treatment differentially modulated *Csls* expression. *CslA1* and *CslD1/3* increased with the increase in the concentration of La (Ш), peaking under 160 mg L^−1^ (*p* < 0.05), while *CslA2~CslA9*, *CslH1/2*, and *CslD2* showed an initial increase followed by a decrease, with most reaching higher levels under 20~80 mg L^−1^ La (Ш) (*p* < 0.05), except for *CslA7*. Notably, the expression pattern of *MSR1* mirrored that of *CslA2*~*CslA9,* with *MSR1.2* exhibiting higher expression under 20~80 mg L^−1^ La (Ш) (*p* < 0.05).

Interestingly, ADP-glucose pyrophosphorylase (*AGP*) biosynthetic genes exhibited a decline to various degrees under La (Ш) treatment. AGP is the first key regulatory and rate-limiting enzyme in starch biosynthesis [27]. Although 20~160 mg L^−1^ La (Ш) increased KGM levels, the *AGP* gene was downregulated (Figure 4B). Moreover, of the 48 DEGs analyzed, 36 exhibited a normal distribution (Shapiro–Wilk test, *p* > 0.05) and were subsequently included in the Pearson correlation analysis. The results revealed significant positive associations between KGM accumulation and *SuSy*, *INV1/3/5/6*, *HK1/2*, *FPK2*, *GPI3*, *PGM3*, *UGP2*, *GMPP1/4*, *CslA3/4/5/6/7*, *CslH2*, and *MSR1.2* but negative correlations with *PGM1*, *GPI2*, and *AGP1* (Figure 4C). Furthermore, five key genes (*UGP1*, *UGP3*, *GPI3*, *MPI1*, and *CslD3*) were identified from the 48 KGM metabolic pathway genes based on a stringent threshold (|log2 FC| > 3) (Table S5). These results indicate that *UGP*, *GPI*, and *Csls* may play crucial roles in KGM biosynthesis and regulation.

### 3.6. Validation of RNA-Seq Results Using qRT-PCR

**Figure 5:**
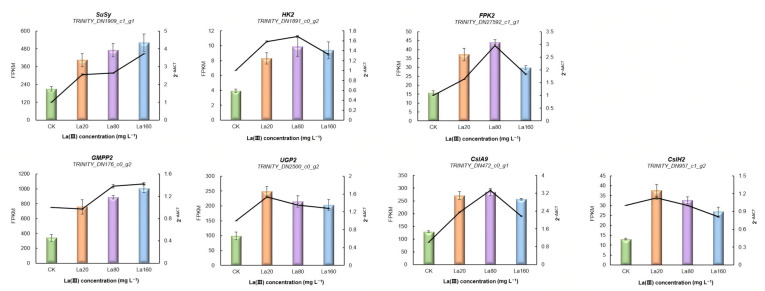
qRT-PCR analysis and FPKM values of KGM biosynthetic genes (means ± SD, *n* = 3). CK: control group. La20: 20 mg·L^−1^ La (Ш) concentration. La80: 80 mg·L^−1^ La (Ш) concentration. La160: 160 mg·L^−1^ La (Ш) concentration.

Seven DEGs from the KGM biosynthetic pathway were randomly selected for qRT-PCR to validate the accuracy of the RNA-Seq data. As shown in Figure 5, the expression of all selected genes was consistent with that determined with RNA-Seq. These findings suggest that the RNA-Seq results are reliable.

### 3.7. Plant Hormone Signal Responses to La (Ш)

**Figure 6:**
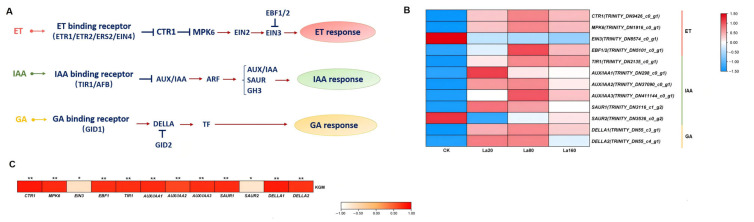
Plant hormone signaling under La (Ш) treatment. (**A**) Hormone signaling, including auxin (IAA), ethylene (ET), and gibberellin (GA). (**B**) Heatmap illustrating expression profiles of DEGs in plant hormone signaling pathways. FPKM values visualized using logarithmic transformation and row-wise normalization. Color scale represents relative expression levels (blue: low; red: high). CK: control group. La20: 20 mg·L^−1^ La (Ш) concentration. La80: 80 mg·L^−1^ La (Ш) concentration. La160: 160 mg·L^−1^ La (Ш) concentration. (**C**) Heatmap depicting FPKM values of plant hormone DEGs according to Pearson correlation with KGM content. Color scale represents relative expression levels (white: low; red: high). * p < 0.05 and ** p < 0.01.

Phytohormones are crucial to plant growth and development. Herein, we focused on secondary metabolites regulated by hormones (Figure 6A). We performed a Pearson correlation analysis and identified six auxin (IAA), four ethylene (ET), and two gibberellin (GA) biosynthetic genes which were significantly associated with KGM accumulation (*p* < 0.05) (Figure 6B). *EIN3* and *SAUR2* expression decreased with the increase in the concentration of La (Ш) (vs. CK) and correlated negatively with KGM content, while other genes displayed an initial increase followed by a decrease, peaking under 20~80 mg L^−1^ La (Ш), and were correlated positively with KGM accumulation (Figure 6C and Table S6).

### 3.8. TF Responses to La (Ш) Treatment

**Figure 7:**
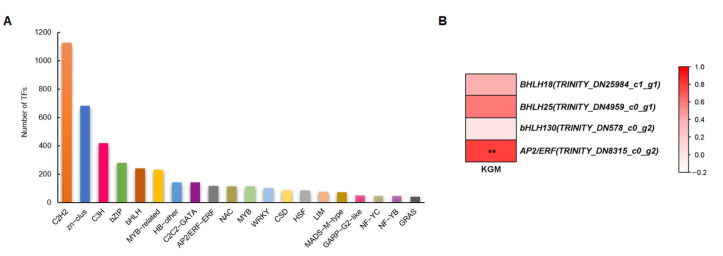
TF responses to La (Ш). (**A**) Top 20 TF genes. (**B**) Heatmap depicting FPKM values of KGM synthesis-related TFs according to Pearson correlation with KGM content (white: low; red: high). ** p < 0.01.

The top 20 TF genes in the RNA-Seq data are shown in Figure 7A. C2H2, zn-clus, C3H, bZIP, and bHLH were the top five classes. The PPI network analysis revealed that the KGM biosynthetic enzymes predominantly interact with transcription factors from the bHLH and AP2/ERF families (Supplementary Figure S1). The Pearson correlation analysis indicated that AP2/ERF expression showed a positive correlation with KGM accumulation (*p* < 0.05); the metabolic regulatory function of AP2/ERF requires further validation (Figure 7B).

## 4. Discussion

The konjac corm developmental process occurs in three sequential stages: the “changing head” stage, the corm expansion stage, and the maturation stage. In the “changing head” stage, the mother corm supplies nutrients for shoot establishment until leaf expansion triggers its senescence, concurrent with daughter corm initiation. During the corm expansion stage, photosynthates from the leaves drive rapid daughter corm growth, with early-stage KGM biosynthesis and the late-stage stabilization of KGM accumulation [25,28,29]. Our previous study demonstrated that a single foliar application of La (Ш) (20~160 mg L^−1^) enhanced the KGM content in *A*. *sinensis* 60 days post-treatment [12]. Therefore, this research project was devised to elucidate the molecular mechanisms underlying La (Ш)-induced KGM biosynthesis. In this study, a foliar application of La (Ш) at varying concentrations (20~160 mg L^−1^) was performed in the early stage of leaf expansion (at the start of daughter corm growth). The KGM content was quantified 14 days post-treatment (the early expansion phase of daughter corms) and 60 days post-treatment (the late expansion phase). The results demonstrate that La (Ш) treatment at concentrations of 20~80 mg L^−1^ significantly enhanced KGM accumulation in the corms compared with CK in both stages (*p* < 0.05) (Figure 1). This indicates persistent growth-promoting activity of La (Ш), which stimulates KGM production throughout the corm enlargement phase.

We further conducted a comprehensive transcriptome profiling of samples treated with La (Ш) at varying concentrations (20~160 mg L^−1^) to elucidate the molecular mechanisms underlying the La (Ш)-mediated regulation of konjac glucomannan (KGM) biosynthesis. In this study, based on the established KGM biosynthetic pathway [25], we identified 48 La (Ш)-responsive genes encoding 15 functional enzymes associated with KGM synthesis, including *SuSy*, *INV*, *FPK*, *HK*, *UGP*, *GMPP*, *CslA*, *CslD*, *CslH*, *GPI*, *MPI*, *PMM*, *PGM*, *MSR1*, and *AGP*. There were seven genes (*Susy*, *MPI1*, *PGM3*, *GMPP2*, *CslA1*, *and CslD1/3*) whose expression increased under different concentrations (20~160 mg L^−1^) of La (Ш), peaking at 160 mg L^−1^ La (Ш) (*p* < 0.05). A total of 25 genes, including *INV1/2/3/4/6*, *HK1*, *FPK2*, *GMPP 1/4/5*, *UGP1/2*, *CslA2~9*, *CslH1/2*, *CslD2*, *GPI3*, and *MSR1.2*, were more highly expressed under 20~80 mg L^−1^ La (Ш). Among them, the expression of 9 genes (*INV2/3*, *GMPP5*, *UGP2,GPI3*, *CslA4/8*, *and CslH1/2*) peaked under 20 mg L^−1^ and that of 6 genes (*INV1/4/6*, *FPK2*, *GMPP1*, *and CslA2*) under 80 mg L^−1^ La (Ш) (*p* < 0.05); further, 10 genes (*HK1*, *GMPP4*, *UGP1*, *CslA3/5/6/7/9*, *CslD2*, and *MSR1.2*) were highly expressed under both 20 mg L^−1^ and 80 mg L^−1^ (*p* < 0.05). These findings show that terminal genes (*Csls*, *GMPP*, *UGP*, and *MSR1*) were more responsive to 20~80 mg L^−1^ La (Ш), which suggests that La (Ш) primarily enhances KGM accumulation by activating terminal pathway genes. Notably, *UGP1*, *UGP3*, and *CslD3* showed a log2FC > 3, further supporting the functional importance of *UGP* and *Csls* as key enzymes in KGM biosynthesis [25,30].

Csls, belonging to the glycosyltransferase GT2 family, are key enzymes that catalyze the synthesis of glycans [26]. *CslAs*, which originate from independent endosymbiosis in green algae [26,31], and *CslDs,* which form a sister clade with CesAs, are known to be involved in the biosynthesis of mannan and glucomannan [32,33,34]. In this study, 14 genes were annotated to *Csls*: 9 genes to *CslA*, 3 genes to *CslD*, and 2 genes to *CslH*. Among the *CslAs* (except for *CslA1*), *CslD2* and *CslH1/2* were more highly expressed under 20~80 mg L^−1^ La (Ш) (*p* < 0.05), which suggests that La (Ш) increased KGM content by upregulating *CslA* gene expression. *CslHs,* as a member of the hemicellulose synthase subfamily [26,34], may share functional similarities with *CslA* and *CslD* in promoting glucomannan biosynthesis, but its function requires further experimental validation. UGP, a key enzyme involved in KGM biosynthesis, catalyzes the reversible production of UDP-Glc and Glc-1-P [35]. The overexpression of *DoUGP* modulated the KGM biosynthetic pathway, upregulating *CslA* expression in the stems of *Dendrobium officinale* and increasing KGM content [36]. *MSR1* plays a role in KGM biosynthesis [37]. *AtMSR1* acts as an enhancer of *AtCslA2*, co-catalyzing KGM biosynthesis [38]. Thus, La (Ш) may directly upregulate *CslA* expression and indirectly enhance *UGP* and *MSR1* expression, ultimately promoting KGM accumulation.

Additionally, Glc-1-P is a shared precursor of starch and KGM biosynthesis [25]. It is converted into UDP-Glc by SuSy or UGP for KGM synthesis, whereas it is metabolized into starch by ADP-Glc in *AGP* catalysis. AGP is the first rate-limiting enzyme in starch biosynthesis, and its expression level is positively correlated with starch content in plants such as *A. muelleri*, potato, and rice [39,40,41,42]. In *A. muelleri*, *AmAGP* was highly expressed in corm under high-starch conditions and little expressed in petioles under low-starch conditions, and its expression was decreased in 150-day corm, while KGM content increased throughout the development period [40]. Antisense-AGP transgenic plants exhibited significantly reduced starch levels, whereas sucrose and glucose levels were increased in potato tubers [42]. AGP was found to be involved in starch production and indirectly regulate sugar formation. La (Ш) at 20~160 mg L^−1^ enhanced KGM content but suppressed *AGP* expression, indicating that La (Ш) may inhibit AGP activity, thereby promoting KGM biosynthesis.

The expression of well-known phosphorylate isomerases (GPI, MPI, PMM, and PGM) is regulated by the plant species, substrate concentration, temperature, and metal ions [43,44]. In this study, the expression of GPI, MPI, PMM, and PGM varied with different La (Ш) concentrations. KGM production was positively correlated with *GPI3* but was negatively correlated with *PGM1* and *PMM2*, and the expression of all *PMM* genes declined with La (Ш) treatment. The overexpression of PGM caused an increase polysaccharide content in *Ganoderma lucidum* [45], and *DoPMM* expression in *A. thaliana* lines induced higher polysaccharide levels than in the wild-type [46]. Conversely, *PGM/PMM* knockout in algae increased Glc-1-P [47]. Elevated Glc-1-P levels promoted UGP activity and polysaccharide synthesis in *Ganoderma lucidum* [35]. Low temperatures caused UGP negative cooperativity toward Glc-1-P in potato [48]. Considering the reversibility and complexity of these reactions and the diverse sources of Glc-1-P and Man-1-P, the roles of *PGM1* and *PMM2* in KGM biosynthesis need further validation.

Apart from the KGM biosynthetic genes, transcription factors may exhibit more comprehensive and indispensable regulatory roles across metabolic pathways. In this study, Apetala2/ethylene-responsive factor (AP2/ERF) and basic helix–loop–helix (bHLH) were closely related to KGM biosynthesis. bHLH plays important roles in plant secondary metabolism. In *A. thaliana*, bHLH proteins interact with myeloblastosis (MYB) and WD40 to form a MYB-bHLH-WD complex, activating several genes related to anthocyanin biosynthesis and resulting in anthocyanin production [49]. MYB6/bHLH13-AbSUS2 is involved in sugar metabolism in *Abies beshanzuensis* [50]. Our Pearson correlation analysis indicated a potential positive association between AP2/ERF expression and KGM contents (*p* < 0.05). AP2/ERF is implicated in diverse biological processes, such as plant growth, development, and responses to hormone and environmental stress [51]. Based on sequence similarities and the number of AP2/ERF domains, the AP2/ERF superfamily can be divided into four categories: AP2, ERF, related to ABI3/VP1 (RAV), and Soloist [51,52]. The ERF family is further divided into two subfamilies, ERF and dewater-responsive element binding (DREB), both of which only contain one AP2/ERF domain and are essential regulators of the responses to biotic and abiotic stress [53]. The overexpression of *CitERF16* in *Citrus reticulata* callus significantly induced *CitSWEET11d* expression and increased sucrose production, indicating that *CitERF16* acts as a positive regulator [54]. AP2/ERF interacts with hormones such as ET, GA, and abscisic acid (ABA) [55,56,57]. During jujube fruit ripening, *ZjERF54* and *DREB39* emerged as key positive regulators, while *ZjERF25* and *ZjERF36* functioned as ripening repressors subjected to 100 μL L^−1^ ET treatment [55]. Similarly, *ZmEREB156* could be stimulated with sucrose and ABA, resulting in starch biosynthesis in maize (*Zea mays*) [51].

Phytohormones play pivotal roles in plant growth and development. As secondary signals, they can initiate a series of signaling events that ultimately induce responsive genes [58]. The correlation analysis of the FPKM values of the DEGs from five phytohormone signaling pathways (ET, GA, IAA, CTK, and ABA) and KGM accumulation allowed us to identify 13 genes from the ET, GA, and IAA biosynthetic pathways which were significantly associated with KGM accumulation, indicating that the La (Ш)-induced phytohormone biosynthetic pathways are involved in KGM biosynthesis. In the absence of ET, *CTR1* binds to *ET* receptors, suppressing downstream genes, while in the presence of ET, *CTR1* activates *EIN2*, promoting *EIN3* accumulation and the activation of downstream genes [56,59]. *EIN3,* a key TF for ET-responsive genes, is negatively regulated by *EBF1/2* [59]. Furthermore, the SIMKK-MPK6 pathway interacts with *CTR1* and positively stimulates ET signaling [59]. Among the four ET biosynthetic genes (*CTR1*, *MPK6*, *EIN3*, and *EBF1/2*), *EIN3* showed a significantly negative correlation with KGM accumulation. However, KGM accumulation showed a significant positive correlation with *DELLA,* which is a key negative regulator of the GA pathway. GA stimulates plant growth by overcoming the growth-restraining effects of the DELLA proteins, a group of nuclear growth repressors [60,61]. Previous studies have shown that DELLAs interact with the DNA-binding domains of EIN3 or EIN3-LIKE 1 (EIL1) in the GA signal transduction pathway [62]. In addition, the overexpression of *CBF1* increased both *DELLA* gene expression and protein deposition in *A. thaliana*, suggesting that *CBF1* transcriptionally modulate DELLA accumulation [63]. Collectively, La (Ш) promoted KGM biosynthesis through interactions between AP2/ERF and the biosynthetic genes (*EIN3* and *DELLA*) involved in the ET and GA signaling pathways (Figure 4A). While the transcriptomic data suggest potential metabolic pathway regulation, this hypothesis requires validation based on integrated metabolomics for the establishment of direct links between gene expression patterns and metabolite accumulation. Furthermore, the targeted CRISPR-Cas9 knockout of the candidate genes would provide definitive functional evidence and clarify their roles in the biosynthetic network.

## 5. Conclusions

In this study, 20~80 mg L^−1^ La (Ш) concentrations promoted KGM accumulation. With transcriptomics, we identified 21,047 DEGs between CK and the groups treated with 20, 80, and 160 mg L^−1^ La (Ш) concentrations enriched in carbohydrate and glycan metabolism. A total of 20 key biosynthetic genes (*SuSy*, *INV1/3/5/6*, *HK1/2*, *FPK2*, *GPI3*, *PGM3*, *UGP2*, *GMPP1/4*, *CslA3/4/5/6/7*, *CslH2*, and *MSR1.2*) were markedly correlated with KGM accumulation. Additionally, 12 hormone-related DEGs, including 4 ET-related genes (*CTR1*, *MPK6*, *EIN3*, and *EBF1/2*), 7 IAA-related genes (*TIR1*, *AUX/IAA1-3*, and *SAUR1-2*), and 2 GA-related genes (*DELLA1-2*), were associated with KGM levels. Moreover, the bHLH and AP2/ERF TF families were found to be closely related to the biosynthesis of KGM, and there was a significant positive correlation between AP2/ERF transcript abundance and KGM accumulation. These findings indicate that moderate La (Ш) exposure appears to activate the transcriptional networks involved in KGM biosynthesis and hormonal signaling, with the concurrent upregulation of the associated TFs potentially driving KGM accumulation in this system. Notably, this study provides a genetic basis for high-quality konjac breeding and insights for rational La (Ш) application in commercial cultivation.

## Figures and Tables

**Table 1:** Summary of RNA-Seq analysis data of *A. konjac* corms exposed to different La (Ш) treatments.

Samples	Raw Reads (Mb)	Raw Bases (Gb)	Clean Reads (Mb)	Aligned Reads (%)	Q30 (%)	GC (%)
CK.1	21.47	6.44	17.90	83.38	92.83	51.03
CK.2	21.18	6.35	16.63	78.52	92.61	49.24
CK.3	24.38	7.31	20.51	84.15	92.69	49.69
La20.1	19.75	5.93	15.83	80.11	91.68	50.94
La20.2	21.27	6.38	17.43	81.95	92.42	52.96
La20.3	23.46	7.04	19.49	83.07	91.97	52.05
La80.1	25.72	7.72	21.21	82.45	91.47	51.93
La80.2	25.42	7.63	20.46	80.48	92.32	52.00
La80.3	24.52	7.35	20.55	83.83	92.15	52.28
La160.1	21.22	6.36	17.55	82.73	92.01	52.52
La160.2	31.71	9.51	25.11	79.17	92.36	52.75
La160.3	29.67	8.90	23.45	79.06	92.23	51.24

## Data Availability

All data generated or analyzed in this study are included in the main text and its Supplementary Materials.

## Supplementary Materials

The following supporting information can be downloaded at: https://www.mdpi.com/article/10.3390/biology14070849/s1, Table S1: Primer Sequences for qRT-PCR; Table S2: KGM contents in La (Ш)-treated corms in defined expansion stages; Table S3: Summary of functional annotations from six public protein databases (COG, GO, KEGG, Pfam, Swiss-Prot, and Nr); Table S4: Multiple comparison analysis of FPKM values of DEGs in KGM biosynthetic pathway and their Pearson correlation coefficients with KGM contents; Table S5: |log2 FC| > 3 of DEGs in KGM biosynthetic pathway under La (Ш) treatment; Table S6: FPKM values of DEGs involved in plant hormone signaling pathways and their Pearson correlation with KGM contents; Figure S1: Protein–protein interaction network between konjac glucomannan (KGM) biosynthetic enzymes and transcription factors under La (Ш) treatment.

## Abbreviations

The following abbreviations are used in this manuscript:

La (III)	lanthanum
DEG	differentially expressed gene
TF	transcription factor
KGM	konjac glucomannan
*A. konjac*	*Amorphophallus konjac*
PPI	protein–protein interaction
Suc	sucrose
Fru	fructose
Glc	glucose
UDP-Glc	UDP-glucose
GDP-man	GDP-mannose
Fru-6-P	fructose-6-phosphate
Fru-1-P	fructose-1-phosphate
Man-6-P	mannose-6-phosphate
Man-1-P	mannose-1-phosphate
Glc-1-P	glucose-1-phosphate
Glc-6-P	glucose-6-phosphate
SuSy	sucrose synthase
INV	invertase
PGI	phosphoglucose isomerase
PGM	phosphoglucomutase
PMI	phosphomannose isomerase
PMM	phosphomannomutase
GMPP	GDP-mannose pyrophosphorylase
UGP	UDP-glucose pyrophosphorylase
AGP	ADP-glucose pyrophosphorylase
FRK	fructokinase
HK	hexokinase
Csls	cellulose synthase-like
MSR1	mannan synthesis-related 1
