# Supplementary material for: Glucomannan Accumulation Induced by Exogenous Lanthanum in Amorphophallus konjac: Insights from a Comparative Transcriptome Analysis
Source: Biology (Basel). 2025 Jul 11;14(7):849. doi: 10.3390/biology14070849 (PMC12292675; doi:10.3390/biology14070849)
Supplement: Supplementary file 1 [file biology-14-00849-s001.zip › biology-3715097-supplementary.pdf]

Supplementary Materials

Table S1. Primer Sequences for qRT-PCR

Primer name	Forward primer(5′→3′)	Reverse primer(5′→3′)
EF-1α	ACAAGATGAGGAGCAGGG	GGTGATAAGGACACGAGA
SuSy(TRINITY_DN1909_c1_g1)	GGGACTTACCGTACAACTCAAC	AGGAGCAAGCCCATCATATTC
HK2(TRINITY_DN1891_c0_g2)	GACAAGGGAGCACAAGGAAA	ACGAGGTCAACAACCTCAATC
FPK2(TRINITY_DN27592_c1_g1)	AGAGAGAGAGAGAGCGCATAA	GACCAACCAGCCGGATTT
GMPP2(TRINITY_DN176_c0_g2)	GATGGTGTGTTCTGCATCCT	CGGCTGCTCTTCTTGATTCT
UGP2(TRINITY_DN2500_c0_g2)	GAGGCCATCAACCCTAACAA	CCACTTGCTTGGGAGAGAAT
CslA9(TRINITY_DN472_c0_g1)	CAGCAGGCAACGTACAGTAT	TGGGTATGGAGGAGGATTGA
CslH2(TRINITY_DN957_c1_g2)	GCAGACATTGTAGGAGGAGAAG	AAAGACACTTGTGTGAGGAGAG

Table S2. KGM content in La(III) treated corms at defined expansion stages (Means ± SD, n = 3)

	CK	La20	La80	La160
14- days post-treatment	3.70±0.08c	4.42±0.18b	5.14±0.17a	3.86±0.37c
60- days post-treatment	9.56±0.10b	12.57±0.93a	13.15±1.46a	11.25±1.05ab

Different lowercase letters indicate significant differences between treatments (Duncan's multiple range *P < 0.05*); **p < 0.05, **p < 0.01* (Pearson's correlation)

Table S3. Summary of functional annotations from six public protein databases (COG, GO, KEGG, Pfam, Swissprot and Nr)

Anno_Database	Annotated_Number	300<=length<1000	length>=1000
COG_Annotation	100765	66919	33846
GO_Annotation	142047	88643	53404
KEGG_Annotation	181135	114067	67068
Pfam_Annotation	163578	103752	59826
Swissprot_Annotation	149376	92821	56555
Nr Annotation	189287	120184	69103
All_Annotated	231443	153306	78137

Table S4. Multiple comparison analysis of DEGs FPKM values in the KGM biosynthetic pathway and their Pearson's correlation coefficients with KGM content.

Gene name	CK	La20	La80	La160	Pearson's correlation
SuSy	210.43±15.57c	404.18±44.54ab	470.63±44.6ab	520.86±57.77a	0.61*
INV1	0.69±0.06c	1.83±0.37b	3.3±0.23a	0.28±0.04d	0.66*
INV2	7.44±0.17c	19.64±2.38a	17.2±1.54ab	14.88±1.11b	0.81
INV3	19.42±0.40c	47.9±5.80a	44.87±6.75ab	36.27±4.77b	0.94**
INV4	34.51±7.06b	13.44±3.22c	66.81±11.75a	16.14±4.10c	0.23
INV5	6.97±1.80b	19.02±1.97a	17.49±3.17a	19.43±2.87a	0.77**
INV6	12.48±1.68c	25.49±5.04b	31.66±2.25a	24.33±1.20b	0.74**
INV7	7.48±1.80b	3.54±0.33c	17.23±1.71a	15.09±1.75a	0.23
HK1	67.49±8.90b	171.84±27.78a	182.61±18.66a	34.57±9.04b	0.61*
HK2	3.94±0.23b	8.31±0.78a	9.9±1.33a	9.39±1.12a	0.75**
FPK1	0.06±0.03a	0.13±0.02a	0.35±0.25a	0.29±0.14a	-
FPK2	15.77±1.18d	37.25±3.49b	44.05±1.45a	29.71±1.21c	0.88**
GPI1	71.33±4.95a	36.75±2.84b	35.38±5.72b	30.3±3.65b	-
GPI2	0.62±0.06a	0.35±0.12b	0.24±0.07b	0.2±0.03b	-0.60*
GPI3	0.00±0.00c	1.30±0.19a	0.75±0.05b	0.66±0.13b	0.72**
GMPP1	3.35±0.72c	13.12±0.55a	7.28±0.67b	6.26±0.09b	0.64*
GMPP2	340.94±42.71d	758.91±95.86c	885.96±23.63b	1005.43±55.22a	0.53

GMPP3	60.39±4.78b	123.54±18.17a	45.84±7.68b	127.58±12.32a	0.09
GMPP4	8.95±1.27c	52.23±6.03a	52.57±6.47a	23.37±5.06b	0.81**
GMPP5	23.4±5.92b	43.18±7.75a	12.45±5.05c	8.8±1.71c	0.12
CslA1	0.12±0.02c	0.52±0.15b	0.25±0.07c	0.87±0.12a	0.17
CslA2	0.15±0.02c	0.07±0.002c	3.14±0.28a	1.46±0.30b	-
CslA3	106.08±10.02b	221.01±27.31a	228.41±14.96a	49.35±7.61c	0.63*
CslA4	5.02±2.34c	31.77±6.04a	18.93±3.33b	16.39±2.91b	0.64*
CslA5	57.66±8.19b	132.24±13.3a	130.79±11.78a	33.9±8.20c	0.64*
CslA6	0.63±0.13b	1.64±0.24a	1.53±0.09a	0.26±0.05c	0.65*
CslA7	23.29±8.44b	47.68±3.30a	50.99±6.42a	12.13±1.65c	0.59*
CslA8	0.66±0.17b	2.65±0.20a	0.31±0.03c	0.26±0.02c	-
CslA9	129.74±3.04c	270.93±15.75ab	284.53±13.95a	256.21±3.36b	-
CslD1	0.63±0.04c	1.39±0.10b	1.22±0.03b	2.81±0.49a	-
CslD2	1.00±0.10b	2.33±0.46a	2.12±0.08a	1.09±0.06b	-
CslD3	0.00±0.00d	0.24±0.03b	0.10±0.01c	0.61±0.01a	-
CslH1	16.13±1.56b	32.28±4.09a	3.56±0.10c	7.5±0.66c	-0.03
CslH2	13.1±0.44d	37.55±3.03a	32.69±1.74b	26.88±2.28c	0.76**
UGP1	0.00±0.00c	11.26±1.55a	10.24±0.81a	7.28±0.87b	-
UGP2	99.36±12.79c	248.73±17.47a	214.78±19.52b	203.11±18.51b	0.78**
UGP3	0.00±0.00b	4.53±1.34a	0.82±0.12b	3.36±0.55a	0.39
MPI1	0.00±0.00c	0.31±0.03b	0.26±0.03b	2.01±0.15a	-
MPI2	124.77±10.4a	71.54±10.75b	62.61±12.24bc	49.25±6.23c	-0.51*
PGM1	100.18±13.61a	48.81±5.93b	44.52±4.88b	23.29±2.11c	-0.60*
PGM2	0.39±0.02a	0.18±0.03b	0.11±0.01c	0.06±0.01d	-
PGM3	3.72±0.06c	8.77±1.58b	12.32±2.1a	13.71±0.73a	0.61*
PMM1	0.85±0.07a	0.22±0.03b	0.11±0.01c	0.06±0.01c	-
PMM2	66.95±10.49a	32.57±3.81b	29.04±2.01b	15.52±1.41c	-0.57
AGP1	2.22±0.57a	1.09±0.14b	0.52±0.03b	1.02±0.16b	-0.75**
AGP2	30.9±2.79a	17.38±4.37b	15.5±3.00b	12.49±1.76b	-0.46
MSR1.1	6.92±1.79b	3.16±0.50b	17.17±2.31a	16.09±5.81a	0.12
MSR1.2	19.25±1.42c	42.14±3.91a	41.92±3.21a	33.58±4.07b	0.86**

Data are presented as means ± SD (n = 3). Different lowercase letters indicate significant differences among groups ($p < 0.05$, Duncan's multiple). Pearson's correlation coefficients between DEGs' FPKM values and KGM content are shown, with significance levels denoted as * $p < 0.05$, ** $p < 0.01$. "-" indicates a Shapiro-Wilk test result of $p < 0.05$ (indicating non-normal distribution), with no Pearson's correlation analysis data available.

TableS5 |log2 FC| > 3 of DEGs in KGM biosynthetic pathway under La(III) treatment

Gene name	CK vs La20	CK vs La80	CK vs La160
UGP1	24.59	24.42	6.37
UGP3	22.71	6.23	8.25
CslD3	4.49	3.31	5.86
GPI3	6.82	6.11	5.99
MPI1	4.90	4.61	7.60

Table S6. FPKM values of DEGs involved in plant hormone signaling pathways and their Pearson's correlation with KGM content.

Gene name	CK	La20	La80	La160	Pearson's correlation
CTR1	8.30±1.58	22.59±3.3	28.08±2.95	23.58±2.61	0.85**
MPK6	17.45±2.59	44.3±3.74	53.68±5.01	45.68±7.44	0.76**
EIN3	36.53±5.72	19.94±4.14	19.07±4.37	18.14±3.93	-0.62*
EBF1/2	11.56±1.29	23.25±5.58	49.44±9.64	33.55±2.42	0.74**
TIR1	7.49±1.42	16.11±1.83	21.07±1.96	19.94±4.35	0.74**

AUX/IAA1	9.42±4.93	29.75±10.09	18.84±5.85	18.02±2.42	0.76**
AUX/IAA2	1.89±0.60	43.35±23.03	48.35±8.81	21.55±17.60	0.80**
AUX/IAA3	2.47±0.45	4.61±1.33	6.80±1.90	5.28±1.12	0.74**
SAUR1	1.58±1.29	9.48±3.56	7.89±3.82	5.12±1.21	0.75**
SAUR2	3.42±1.52	0.58±0.17	1.54±1.03	1.84±0.27	-0.68*
DELLA1	8.78±1.51	18.48±1.87	20.18±1.24	17.18±4.57	0.83**
DELLA2	1.90±1.71	5.88±1.65	5.75±0.42	3.68±1.52	0.72**

Data are presented as means ± SD (n = 3). Pearson's correlation coefficients between DEGs' FPKM values and KGM content are shown, with significance levels denoted as * $p < 0.05$, ** $p < 0.01$.

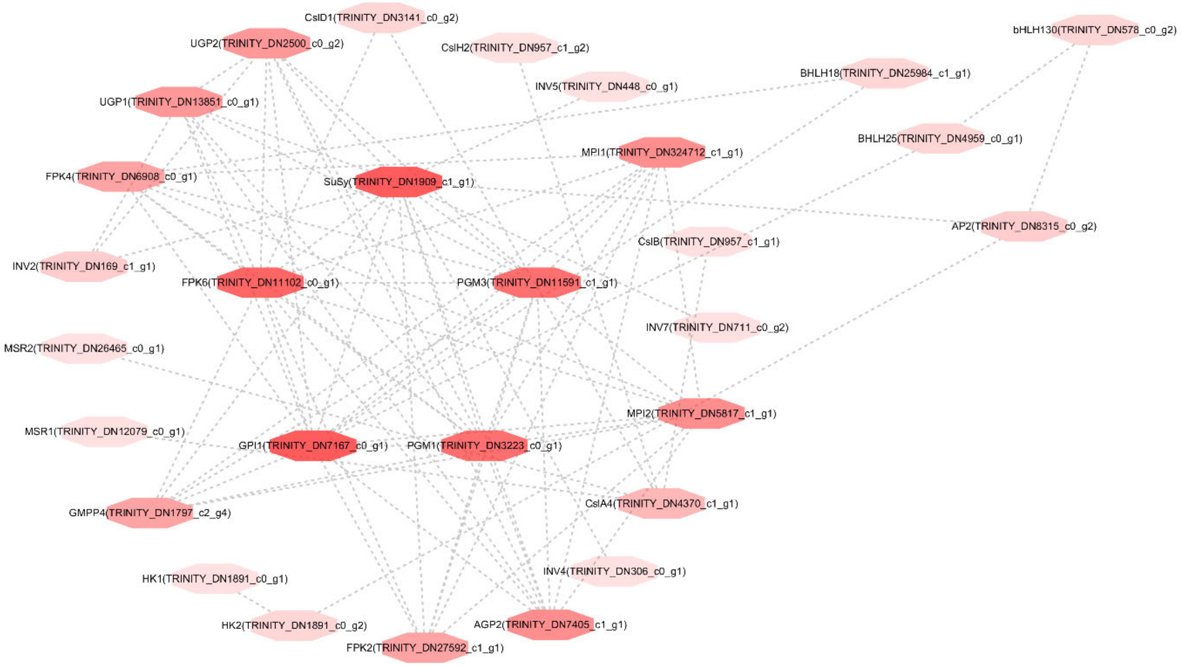

Figure S1 Protein-protein interaction network between konjac glucomannan (KGM) biosynthetic enzymes and its transcription factors under La (III) treatment
